# Mitochondrial Protein Abundance Gradients Require the Distribution of Separated Mitochondria

**DOI:** 10.3390/biology10070572

**Published:** 2021-06-23

**Authors:** Franziska Bollmann, Jan-Niklas Dohrke, Christian A. Wurm, Daniel C. Jans, Stefan Jakobs

**Affiliations:** 1 Department of NanoBiophotonics, Max Planck Institute for Biophysical Chemistry, 37077 Göttingen, Germany; franziska.bollmann@sartorius.com (F.B.); jan-niklas.dohrke@mpibpc.mpg.de (J.-N.D.); c.wurm@abberior-instruments.com (C.A.W.); daniel.jans@mpibpc.mpg.de (D.C.J.); 2 Clinic of Neurology, University of Göttingen, 37073 Göttingen, Germany; 3 Fraunhofer Institute for Translational Medicine and Pharmacology ITMP, Translational Neuroinflammation and Automated Microscopy TNM, 37075 Göttingen, Germany

**Keywords:** image analysis, inner-cellular heterogeneity, nanoscopy, protein distribution, super-resolution microscopy

## Abstract

**Simple Summary:**

Individual mitochondria within a cell can be heterogeneous regarding their physiological and morphological characteristics. Gradients in the abundance of proteins from the perinuclear to the peripheral mitochondria have previously been demonstrated. The molecular mechanisms underlying these gradients are unknown. In this study, we demonstrate, through the example of the protein Tom20, a subunit of the translocase of the mitochondrial outer membrane, that abundance gradients are formed quickly after cell division. Moreover, these gradients require separated mitochondria and intact microtubules. This suggests an active process that positions mitochondria according to their properties within the cell.

**Abstract:**

Mitochondria are highly dynamic organelles that interchange their contents mediated by fission and fusion. However, it has previously been shown that the mitochondria of cultured human epithelial cells exhibit a gradient in the relative abundance of several proteins, with the perinuclear mitochondria generally exhibiting a higher protein abundance than the peripheral mitochondria. The molecular mechanisms that are required for the establishment and the maintenance of such inner-cellular mitochondrial protein abundance gradients are unknown. We verified the existence of inner-cellular gradients in the abundance of clusters of the mitochondrial outer membrane protein Tom20 in the mitochondria of kidney epithelial cells from an African green monkey (Vero cells) using STED nanoscopy and confocal microscopy. We found that the Tom20 gradients are established immediately after cell division and require the presence of microtubules. Furthermore, the gradients are abrogated in hyperfused mitochondrial networks. Our results suggest that inner-cellular protein abundance gradients from the perinuclear to the peripheral mitochondria are established by the trafficking of individual mitochondria to their respective cellular destination.

## 1. Introduction 

Mitochondria are double membrane organelles that have central roles in numerous cellular processes including ATP generation, lipid metabolism, calcium signaling, and apoptosis [1,2,3,4]. In many mammalian cell types, the mitochondria form a dynamic and highly heteromorphic network of tubules. The structure of the mitochondrial network relates to the physiological status of the cell and different cell types may exhibit distinct mitochondrial shapes [5]. A growing body of evidence shows that within a single cell, the individual mitochondria exhibit varying protein contents and ultra-structures [6,7,8,9,10]. Single-cell studies that use live cell markers for matrix pH, mitochondrial membrane potential, Ca^2+^ concentrations, the redox state or reactive oxygen species, also suggested functional heterogeneity between different mitochondria [11,12,13], and even between individual cristae [14]. Increased mitochondrial heterogeneity has been reported in several disease models [15]. 

Previously, it was demonstrated that in several cell types including human skin fibroblasts, the mitochondria close to the nucleus differ from those in the periphery with respect to functional activity and protein content [6]. With the use of STED super-resolution microscopy (nanoscopy), it was shown that several proteins of the inner and outer membrane are more abundant in the perinuclear than in the peripheral mitochondria [6,7]. This demonstrated the existence of inner-cellular protein abundance gradients from the perinuclear to the peripheral mitochondria. 

Intriguingly, mitochondria fuse and divide, and thereby their contents intermix [16,17,18]. It is not known how the heterogeneity between individual mitochondria is established and maintained despite this continuous content mixing.

In this study, using confocal and STED super-resolution microscopy, we demonstrate that the inner-cellular protein abundance gradients are immediately established after cell division, suggesting an active sorting of the mitochondria. We further show that hyperfusion of the mitochondria largely abrogates the gradients, and that an intact microtubule cytoskeleton is required. Together, our data suggest that directed mitochondrial trafficking as well as separated, individual mitochondria are required for the establishment and maintenance of inner-cellular mitochondrial protein abundance gradients.

## 2. Materials and Methods

### 2.1. Cell Culture

Vero cells were cultivated in DMEM with Glutamax and 4.5% (wt/vol) glucose (Invitrogen, Carlsbad, CA, USA), supplemented with 100 U/ml penicillin, 100 μg/ml streptomycin, 1 mM Na-pyruvate, and 10% (vol/vol) FCS (Invitrogen) at 37 °C, 5% CO_2_. 

### 2.2. RNA Interference

For RNA interference, the following plasmid was used: U6-shRNA DRP1 plasmid (target sequence: 5′-TTCAATCCGTGATGAGTATGCTTTTCTTC-3′). Transfections were carried out using Turbofect (Thermo Fisher Scientific, Waltham, MA, USA) according to the manufacturer’s recommendations. After transfection, cells were cultured for 72 h and prepared for microscopy.

### 2.3. Sample Preparation for Fluorescence Microscopy

Immunofluorescence sample preparation was performed, as described previously [6,7].

### 2.4. Fluorescence Microscopy

For confocal microscopy, a TCS SP5 microscope (Leica Microsystems, Wetzlar, Germany), equipped with a 63× oil immersion objective or a 63× water immersion objective, was used. For STED and the corresponding confocal microscopy, a custom-built STED [6] or a STED 775 Quad Scan (Abberior Instruments Göttingen, Germany) microscope was used. 

Time-lapse imaging of Vero cells expressing mtEosFP was performed using a confocal microscope (TCS SP5). Cells were imaged two days after transfection with pcDNA3-mito-EosFPwt plasmid (Mobitec, Göttingen, Germany). During imaging, cells were cultivated in colorless DMEM-Medium at room temperature. Using the FRAP-wizard of the Leica acquisition software, 10 µm × 10 µm regions-of-interest (ROIs) were illuminated with a UV-laser (405 nm diode laser, total of 15 frames), thereby converting the green fluorescent EosFP to its red fluorescent form. Excitation was performed at 488 nm and at 561 nm. Detection was realized sequentially at 495–520 nm and 570–650 nm. Between the images, cells were cultivated in an incubator at 37 °C/5% CO_2_. 

### 2.5. Quantification of the Distribution of Mitochondrial Proteins

**Figure 1:**
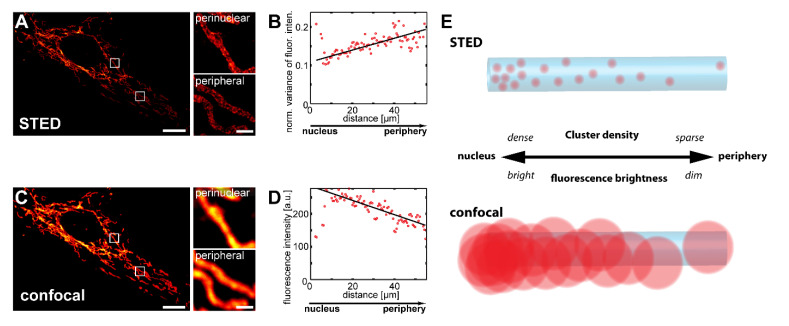
Tom20 forms an inner-cellular abundance gradient, which can be detected with confocal microscopy. (**A**–**D**) STED super-resolution and diffraction-limited confocal microscopy reveal inner-cellular Tom20 abundance gradients. Vero cells were decorated with antiserum against Tom20 and imaged by STED (**A**) or confocal (**C**) microscopy. Small images are magnifications of perinuclear or peripheral mitochondria, as indicated by rectangles in the corresponding overview images. Analysis of inner-cellular Tom20 abundance gradients using the STED (**B**) and the confocal (**D**) image data. Plotted are the averaged normalized local variance values of the fluorescence intensity (**B**) or the averaged fluorescence intensity values (**D**), each from the center of the cell, as shown in (**A**,**C**), to its periphery. Schematic showing the effect of abundance gradients of mitochondrial proteins on the appearance in images recorded with STED and confocal microscopy (**E**). Scale bars: 1 µm (magnifications), 10 µm (overviews).

For the analysis of STED images (used in Figure 1A), an algorithm that calculates the variance of the fluorescence intensity within mitochondria was used, as described in [6].

A similar algorithm was used to semi-automatically detect the mitochondria in the confocal images using MATLAB (Mathworks, Natick, MA, USA). This algorithm analyzes the local fluorescence signals within mitochondria and plots them in relation to the distance to the center of the nucleus. All analyses were performed on maximum intensity projections of confocal sections covering the entire cell along the *z*-axis. In brief, first, individual cells, nuclei, and regions with potentially overlapping mitochondria were singled out by creating manual masks. Then, the mitochondria were automatically selected using binary masks that were created by the application of a (local) isodata threshold. Within these masks, a midline was automatically determined and expanded to define the final masks used for signal intensity analysis. Subsequently, within these mitochondrial masks, the fluorescence signal was determined at each pixel and plotted against its distance to the nucleus. These data points were linearly fitted. The slope of these fits (given in ΔInt/µm) reflects the steepness of the inner-cellular mitochondrial protein abundance gradients. To account for varying cell sizes, we normalized the slope to the size of the cell (the resulting value is given in ΔInt). 

For further validation, an additional analysis of STED images was performed (used in Figure 3E). In short, single clusters were detected using a Laplacian of Gaussian (LoG) filter. Due to the noise sensitivity of the LoG-filter, STED images were denoised in advance using a DenoiSeg filter [19] with all default values (except training epochs = 500). After denoising, a LoG-filter with a sigma of 1.5 pixels (=30 nm) was applied to detect single clusters with a manually curated threshold. Binarization of the denoised images was used to identify mitochondria and the number of detected clusters per mitochondrion area was counted. The resulting cluster density values were plotted against the distance of the center of mass of the individual mitochondria to the center of the nucleus.

### 2.6. Statistical Analysis

All experiments were performed independently at least three times. For the comparison of two groups, statistical significance was evaluated by utilizing two-sample *t* tests using the software Origin (Northampton, MA, USA). 

## 3. Results and Discussion

### 3.1. Confocal Microscopy Enables the Visualization of Inner-Cellular Mitochondrial Protein Abundance Gradients

Using STED super-resolution microscopy, it was previously demonstrated that Tom20 and Tom22, receptors of the translocase of the mitochondrial outer membrane (TOM) complex, as well as the inner membrane protein Mic60 are on average less abundant in peripheral mitochondria than in the perinuclear mitochondria of various mammalian cell types including primary adult human skin fibroblasts [6,7]. With STED microscopy, individual mitochondrial protein clusters could be discriminated and the analysis of the variance of the fluorescence distribution proved to be a sensitive readout for determining variations in cluster densities [6]. 

To verify these findings, we investigated the variations in the Tom20 cluster density in Vero cells, a cell line derived from epithelial cells extracted from an African green monkey. In Vero cells, the mitochondria form a network of branched tubules that are partly interconnected (Figure 1A). In these Vero cells analyzed by STED microcopy, we observed a gradient in the normalized variance of the fluorescence intensity from the cell center to the periphery, indicating an inner-cellular decrease in the Tom20 cluster density, and thus confirming the previous studies (Figure 1A,B). The high spatial resolution attainable with STED microscopy requires small pixel sizes, resulting in relatively long image acquisition times [20]. This makes the imaging of large sample numbers, which are generally required for statistically robust conclusions, challenging. Conventional diffraction-limited confocal microscopy allows for faster imaging, but it cannot resolve the individual Tom20 clusters (Figure 1C). Because the density of protein clusters within a mitochondrion is expected to correlate with the fluorescence signal in immunolabeled samples, even if the individual clusters cannot be resolved, we speculated that diffraction limited microscopy should also allow us to visualize such inner-cellular gradients. To test this idea, we recorded confocal z-stacks and analyzed maximum intensity projections to include all the mitochondria of a cell. Indeed, data analysis of the maximum projections revealed a decrease in the fluorescence signal from the perinuclear to the peripheral mitochondria, fully in line with the corresponding STED data (Figure 1A–E). We found such fluorescence intensity gradients in the majority (>99%; n = 414) of all Vero cells decorated with antiserum against Tom20. 

We conclude that conventional confocal microscopy of Vero cells is a robust and fast alternative to STED recordings for the statistical analysis of inner-cellular mitochondrial protein abundance gradients. 

### 3.2. Mitochondrial Protein Abundance Gradients Are Established Immediately after Cell Division

Using confocal microscopy, we next investigated the cell cycle stages at which the inner-cellular mitochondrial protein abundance gradients are established. Upon cell division, the mitochondrial tubules undergo a massive structural rearrangement involving fragmentation, condensation, and mixing of the network [21,22,23,24]. These morphological changes may influence the analysis of the gradients despite the optical sectioning provided by the confocal microscope, as two axially overlapping mitochondria might add up their fluorescence signals. To avoid an influence of this effect, we relied on images where the overlapping mitochondria were manually excluded for all further analysis.

**Figure 2:**
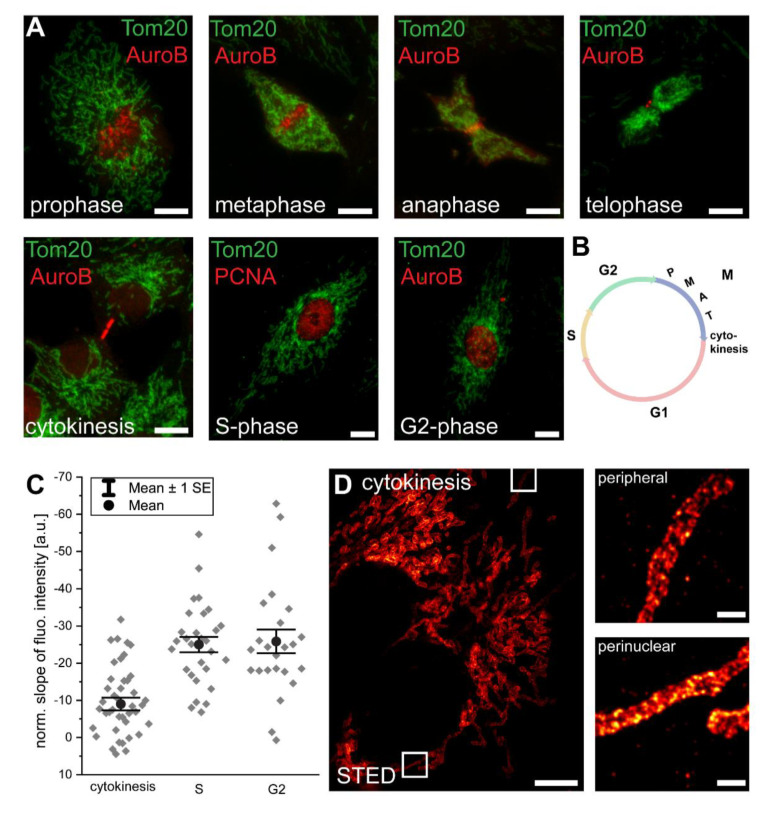
Immediately after mitosis, a Tom20 abundance gradient is generated. (**A**) In a non-synchronized cell culture, cell-cycle stages were assigned using antisera against Aurora B kinase and PCNA. (**B**) Schematic of the cell cycle. (**C**) Analysis of the normalized slopes of the Tom20 fluorescence intensity gradients of cells at cytokinesis, S-phase, or G2-phase. Overlapping mitochondria were excluded from the analysis. Each grey rhomb represents one cell. Black circle: mean. Error bars: standard error of the mean. (**D**) STED microscopy of a cell at cytokinesis, labeled with antiserum against Tom20. Small images are magnifications of perinuclear or peripheral mitochondria as indicated by rectangles in the overview image. Scale bars: 0.5 µm (**D**: magnifications), 5 µm (**D**: overview), 10 µm (**A**).

As for the identification of cell cycle stages in an unsynchronized proliferating cell culture, in addition to the Tom20 immunolabeling, we labeled the cells with antiserum against Aurora B kinase, which marks the kinetochore during mitosis, labels the midbody at cytokinesis, and associates with heterochromatin in G_2_ [22] (Figure 2A,B). Alternatively, we labeled the cells with an antiserum against PCNA, which localizes to replication foci during the S-phase [22] (Figure 2A,B). 

During the transition from metaphase to telophase, the overall mitochondrial mass was contracted to a small volume. This condensation of the mitochondrial network precluded the analysis of potential inner-cellular protein gradients (Figure 2A). We found that immediately after mitosis, at late cytokinesis and just after the distribution of the mitochondria to the two daughter cells, a Tom20 abundance gradient was detectable (n = 42 cells) (Figure 2C). This difference between perinuclear and peripheral mitochondria in cytokinesis was also visible in the STED images of cells decorated with an antiserum against Tom20 (Figure 2D). The initial gradients increased further during the subsequent S- and G_2_-phases (Figure 2C). Notably, we did not observe a hyperfused mitochondrial network at the G_1_-S transition in the Vero cells, which can be very pronounced in some cell types [22], but appears to be absent in others [21]. 

The rapid appearance of a Tom20 intensity gradient immediately after mitosis renders protein synthesis and degradation an unlikely explanation for the generation of the observed gradients. A tempting explanation for the fast generation of mitochondrial protein gradients after the intermixing of mitochondria during mitosis is a directed movement of the mitochondria to their specific localizations in the cell after cell division. Hence, we next addressed the question if inner-cellular trafficking is required for the maintenance of these gradients. 

### 3.3. Disruption of Microtubules Reduces Inner-Cellular Mitochondrial Protein Abundance Gradients

**Figure 3:**
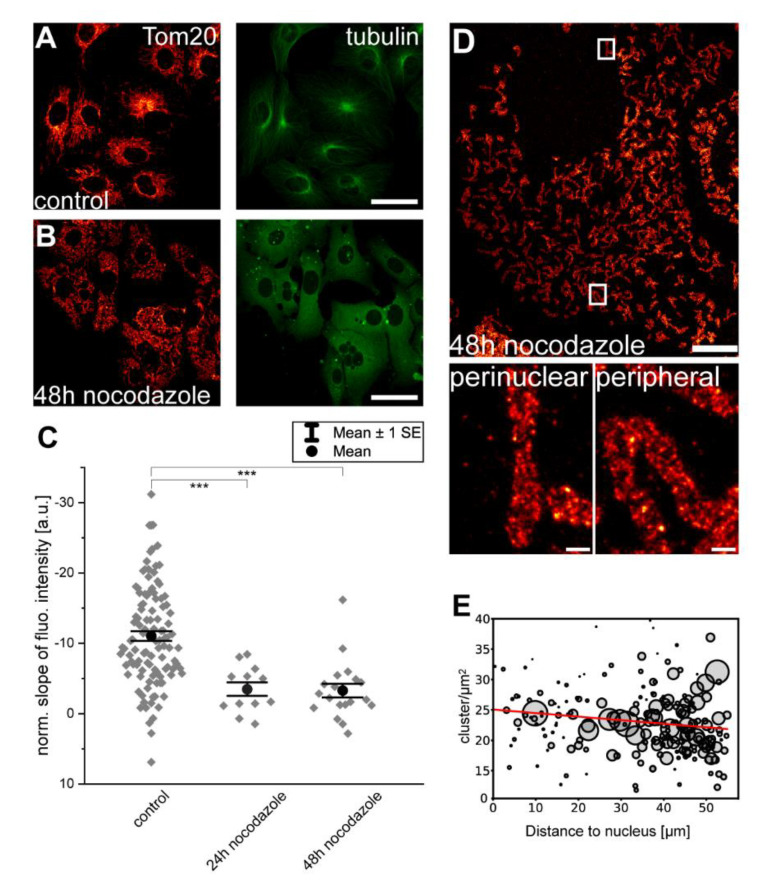
An intact microtubule-cytoskeleton is necessary for the maintenance of inner-cellular Tom20 protein abundance gradients. Cells were mock-treated (**A**) or incubated for 48 h with the microtubule depolymerizing compound, nocodazole (**B**). Cells were labeled with antisera against α-tubulin and Tom20. (**C**) Normalized slopes of the fluorescence intensity gradients of control and nocodazole treated cells decorated with antiserum against Tom20. For the analysis, overlapping mitochondria were excluded. Each grey rhomb represents one cell. Black dot: mean. Error bars: Standard error of the mean. *** *p* = 0.001 (paired *t* test analysis). (**D**) STED image of a cell treated for 48 h with nocodazole and labeled with antiserum against Tom20. Overview image (top) and magnifications of perinuclear mitochondria and peripheral mitochondria from the indicated areas. (**E**) Analysis of the Tom20 cluster density in (**D**). Each sphere represents one mitochondrion within the image. The size of the spheres corresponds to the area of the mitochondrion. Scale bars: 0.5 µm (**D**: magnifications), 10 µm (**D**: overview), and 40 µm (**A**,**B**).

For long-distance trafficking, mitochondria are primarily moved along the microtubules in mammalian cells [25]. We next investigated whether an intact microtubule cytoskeleton is required for the maintenance of inner-cellular mitochondrial protein abundance gradients. To this end, we incubated Vero cells for 24 h or 48 h, with 5 µg/mL of the microtubule depolymerizing substance nocodazole. Subsequently, the cells were labeled with antisera against α-tubulin and Tom20. Whereas the mock-treated control cells exhibited an intact microtubule cytoskeleton, it disappeared entirely and only cytoplasmic fluorescence due to soluble α-tubulin was visible in the nocodazole-treated cells (Figure 3A,B). The disruption of the microtubule cytoskeleton induced a rather equal distribution of the mitochondria over the cell, presumably due to the loss of their attachment to the microtubules. More importantly, we concomitantly observed a strong reduction of the inner-cellular mitochondrial Tom20 abundance gradients using confocal microscopy (24 h: n = 12 cells; 48 h: n = 19 cells) (Figure 3C). This nocodazole-induced change in the distribution of Tom20 is also apparent in the STED super-resolution images (Figure 3D,E). The STED data revealed that although the Tom20 clusters were more numerous in the perinuclear mitochondria than in their peripheral counterparts in mock-treated cells (see also Figure 1A), such a difference was no longer apparent in the nocodazole-treated cells. We also asked if mitochondrial respiration is a prerequisite for the observed Tom20 abundance gradients. To this end, we incubated Vero cells for 48 h with 10 µM of the ATP synthase inhibiting substance oligomycin. The inhibition of the ATP synthase led to the fragmentation of the mitochondrial network (Figure S1A,B). However, analysis of the mitochondrial Tom20 abundance gradients revealed no significant differences between mock- and oligomycin-treated cells (Figure S1C). This indicates that mitochondrial respiration is not a prerequisite for the Tom20 abundance gradients.

We conclude that the depletion of the microtubule cytoskeleton strongly reduces the inner-cellular Tom20 abundance gradients. This suggests that microtubule-aided trafficking of mitochondria is required for the establishment or maintenance of inner-cellular abundance gradients.

### 3.4. Inner-Cellular Mitochondrial Protein Gradients Result from Protein Abundance Differences between Separated Mitochondria

**Figure 4:**
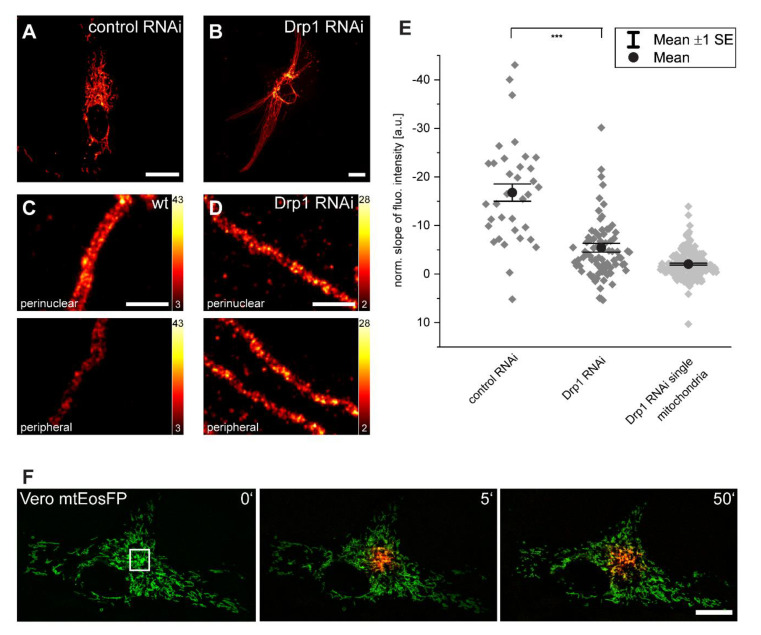
Inhibition of mitochondrial fission reduces inner-cellular Tom20 protein abundance gradients. (**A**,**B**) RNAi-mediated knockdown of Drp1 leads to the elongation of mitochondria and the hyperfusion of the mitochondrial network. (**C**,**D**) Representative STED images of perinuclear and peripheral mitochondria in control (**C**) and Drp1-knockdown (**D**) cells labeled with a Tom20 antibody. Normalized slopes of the fluorescence intensity gradients of control and of Drp1-knockdown cells, and of single mitochondria in Drp1-knockdown cells decorated with antiserum against Tom20. (**E**) For the analysis, overlapping mitochondria were excluded. *** *p* = 0.001 (paired t test analysis). Each dark grey rhomb (control RNAi and Drp1 RNAi) represents one cell; each light grey rhomb (Drp1 RNAi single mitochondria) represents one mitochondrion. Black dot: mean. Error bars: standard error of the mean. Live-cell confocal imaging of Vero cells expressing mitochondrial matrix targeted mtEosFP. (**F**) At t = 0 min in the indicated area, the green fluorescent mtEosFP was photoconverted into its red fluorescent form. Subsequently, the spreading of the photoconverted mtEosFP was recorded. Scale bars: 1 µm (**C**,**D**), 20 µm (**A**,**B**,**F**).

In Vero cells, the mitochondria form rather loose networks with many branched tubules that are not connected to other mitochondrial tubules (Figure 4A). Hence, the entire mitochondrial compartment does not form a continuous luminal network at a given time point. In fact, even for more intertwined mitochondrial networks in other cell types, it has been demonstrated that the mitochondrial networks are often not luminally continuous [26]. 

In order to answer the question of whether physical separation of individual mitochondria in a cell is a requirement of protein abundance gradients, we inhibited mitochondrial fission by down-regulating the large GTPase Drp1 with RNA interference (RNAi) [27]. As a result, the cells exhibited hyperfused and elongated mitochondria that span the entire cell (Figure 4B). 

We immunolabeled control RNAi cells and Drp1 RNAi cells with an antibody against Tom20 (Figure 4A–C). Using STED microscopy, we observed no difference in the Tom20 distribution between the perinuclear and peripheral mitochondria in Drp1 RNAi cells, whereas a difference in the cluster density was obvious in most wild type cells (Figure 4C,D). To further substantiate this finding, we analyzed the abundance of Tom20 based on the distance from the nucleus using confocal microscopy; the slopes of the fluorescence intensity was subsequently calculated (Figure 4E). We observed a significant reduction of the average slopes of the gradients in the Drp1 KD cells (n = 79) as compared to wild type cells (n = 35), suggesting that the physical separation of mitochondria is required to maintain mitochondrial protein abundance gradients.

The effect of mitochondrial connectivity on the protein abundance gradients was even more obvious in the individual mitochondria of the Drp1 RNAi cells (n = 188) that were outstretched from a perinuclear to a peripheral cellular region. In these very long outstretched mitochondria, the Tom20 intensity gradients were further reduced so that they were almost no longer detectable (Figure 4E).

Together, these data show that inner-cellular mitochondrial protein abundance gradients require separated mitochondria. Consequently, the existence of inner-cellular gradients must be due to differences between adjacent mitochondria. Differences between adjacent mitochondria might be unexpected, as it is well known that mitochondria are highly dynamic organelles. They fuse and divide, which facilitates the exchange of proteins between fusing mitochondria [5,28,29]. These dynamics might therefore be expected to oppose stable inner-cellular protein abundance gradients, leading to the question of how these gradients are possible. To address this question, we quantified the dynamic connectivity within the mitochondrial network in the Vero cells. To this end, the photoconvertible fluorescent protein EosFP was targeted to the mitochondrial matrix (mtEosFP). EosFP emits green fluorescence (516 nm) and is irreversibly converted into a red (581 nm) fluorescent form upon near-UV irradiation at 405 nm [30]. We photoconverted mtEosFP in a spatially confined area of the living cells. Fluorescent proteins targeted to the mitochondrial matrix diffuse almost unhindered in the mitochondrial matrix of continuous mitochondria [28,29]. Hence, the spread of the red fluorescent signal reflects the level of connectivity in the mitochondrial network. We followed the spreading of the red fluorescent signal in live Vero cells for up to 50 min (Figure 4F). Despite frequent fusion and fission events, we observed only a minor spreading of the red fluorescent signal out of the photoconverted area. A similar observation has been reported for HeLa cells [30], although other studies showed a faster equilibration of mitochondrial proteins across the entire network in cultivated cells [26,31], possibly pointing to differences in the connectivity of the mitochondria in different cell types. 

We conclude that although mitochondrial dynamics presumably opposes mitochondrial protein abundance gradients, it does not preclude such gradients. At least in Vero cells, the mitochondrial movement and fusion are rather local and thus do not result in a rapid equilibration of mitochondrial protein contents across the entire cell.

## 4. Conclusions

It has been demonstrated that gradients in the mitochondrial membrane potential and the cellular ATP to ADP ratio from the perinuclear to peripheral cellular regions exist [6,32]. This may reflect a higher demand for ATP around the nucleus than in the periphery of the cell. The inner-cellular mitochondrial protein abundance gradients are fully in line with theses activity gradients. We have shown here that microtubules, which are required for effective mitochondrial trafficking, as well as the physical separation of mitochondria, are prerequisites for the mitochondrial protein abundance gradients. Since these gradients are formed quickly after cell division, it is tempting to assume a directed movement of the mitochondria to their specific localizations in the cell according to their properties. Future investigations of cells with high energy demands at the cell periphery such as polarized cells—e.g., neurons or migrating cells—may further clarify the role of targeted mitochondrial trafficking according to their protein content. Intriguingly, it has been shown that mitochondrial dynamics is affected in several diseases such as cancer and neurodegenerative disorders [33,34]. Hence, in some of these cases, the distribution of mitochondria in the cell according to their properties may be disturbed. This might yet be another contributor to aberrant cell function in these diseases.

## Data Availability

All original data will be available from the corresponding author upon reasonable request.

## Supplementary Materials

The following are available online at https://www.mdpi.com/article/10.3390/biology10070572/s1, Figure S1: Mitochondrial respiration is no prerequisite for inner-cellular Tom20 protein abundance gradients. Cells were mock treated (**A**) or incubated for 48 h with the ATP synthase inhibiting compound oligomycin (**B**). Cells were labeled with antiserum against Tom20. (**C**) Normalized slopes of the fluorescence intensity gradients of control and oligomycin treated cells decorated with antiserum against Tom20. For the analysis, overlapping mitochondria were excluded. Each grey rhomb represents one cell. Black dot: mean. Error bars: Standard error of the mean. Scale bars: 40 µm (**A**,**B**).
