# Supplementary material for: Mitochondrial Protein Abundance Gradients Require the Distribution of Separated Mitochondria
Source: Biology (Basel). 2021 Jun 23;10(7):572. doi: 10.3390/biology10070572 (PMC8301041; doi:10.3390/biology10070572)
Supplement: Supplementary file 1 [file biology-10-00572-s001.zip › biology-1244569-supplementary.pdf]

Supplementary Information

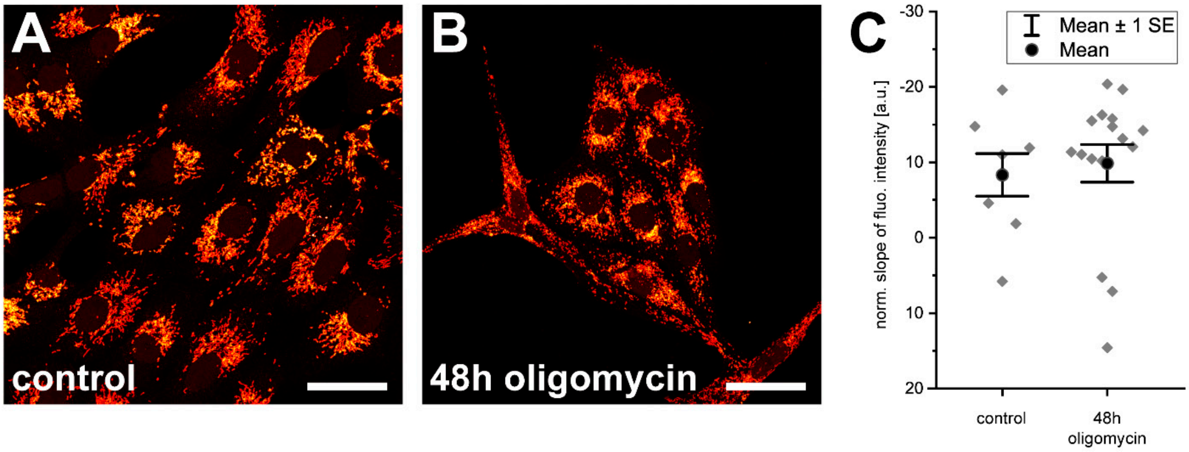

Figure S1. Mitochondrial respiration is no prerequisite for inner-cellular Tom20 protein abundance gradients. Cells were mock treated (**A**) or incubated for 48 h with the ATP synthase inhibiting compound oligomycin (**B**). Cells were labeled with antiserum against Tom20. (**C**) Normalized slopes of the fluorescence intensity gradients of control and oligomycin treated cells decorated with antiserum against Tom20. For the analysis, overlapping mitochondria were excluded. Each grey rhomb represents one cell. Black dot: mean. Error bars: Standard error of the mean. Scale bars: 40 µm (A,B).
